# Antidiabetic effects of *Mangifera indica* Kernel Flour‐supplemented diet in streptozotocin‐induced type 2 diabetes in rats

**DOI:** 10.1002/fsn3.348

**Published:** 2016-02-17

**Authors:** Emmanuel A. Irondi, Ganiyu Oboh, Afolabi A. Akindahunsi

**Affiliations:** ^1^ Department of BiochemistryFederal University of TechnologyP.M.B. 704Akure340001Nigeria; ^2^ Biochemistry UnitDepartments of Biosciences and BiotechnologyKwara State UniversityMaleteP.M.B. 1530IlorinNigeria

**Keywords:** Hyperglycemia, hyperlipidemia, *Mangifera indica* kernel, plasma electrolytes, type 2 diabetes

## Abstract

Our previous report showed that *Mangifera indica* kernel flour (MIKF) is a rich source of pharmacologically important flavonoids and phenolic acids; and that its methanolic extract inhibits some key enzymes linked to the pathology and complications of type 2 diabetes (T2D) in vitro. Hence, this study evaluated the antidiabetic effects of 10% and 20% MIKF‐supplemented diets in T2D in rats. T2D was induced in rats using a high‐fat diet (HFD), low‐dose streptozotocin (HFD/STZ) model, by feeding the rats with HFD for 2 weeks followed by single dose administration of STZ (40 mg/kg body weight, intraperitoneally). The diabetic rats were later fed the MIKF‐supplemented diets, or administered with metformin (25 mg/kg b.w.) for 21 days; the control rats were fed basal diet during this period. Intake of the MIKF‐supplemented diets resulted in significant (*P* < 0.05) improvement in the fasting blood glucose, hepatic glycogen, glycosylated hemoglobin, lipid profile, plasma electrolytes, hepatic and pancreatic malonaldehyde, and the liver function markers of the diabetic rats, compared with the diabetic control rats. The ameliorative effect of 20% MIKF‐supplemented was comparable (*P* > 0.05) with that of metformin administration in the diabetic rats. It is concluded that *M. indica* kernel flour has antidiabetic effects in T2D rats, and could therefore be a promising nutraceutical therapy for the management of T2D and its associated complications.

## Introduction

According to World Health Organisation (WHO), about 350 million people have diabetes globally, compared to 153 million in 1980, and 90% of these cases are Type 2 diabetes (T2D) (Danaei et al. 2011). This translates to about 56% increase in global diabetes prevalence from 1980 to date. With this current global prevalence and a projection that 439 million people will have T2D by 2030 (International Diabetes Federation 2009), the disease is rising at an alarming rate and may assume an epidemic proportion in the near future, if research is not intensified more on preventive than curative strategies. The challenge posed by this dreadful prevalence rate of T2D is further compounded by the complexity and complications of the disease. Hence, despite advances in modern medicine and a huge capital investment, T2D remains a major global health problem, as it has no satisfactory therapy in terms of safety and efficacy. As a metabolic disorder characterized by chronic hyperglycemia, resulting from insulin resistance and loss of pancreas *β*‐cell function (Laakso 2001), T2D is often associated with dyslipidemia (Ahmed and Goldstein 2006).

It is heart‐warming to know that the incidence of T2D can be reduced by half among individuals at high risk through lifestyle and pharmacologic interventions (Li et al. 2008). But incidentally, the currently available antidiabetic drugs for the treatment of T2D have certain drawbacks ranging from adverse side effects and development of resistance to lack of responsiveness in many diabetic patients, in addition to their inability to effectively control the hyperlipidemia that is frequently associated with the disease (Zia et al. 2001). Interestingly, the use of dietary therapy offers hope for a more effective and safer preventative and management strategy for T2D. This has been proven by epidemiological studies that have consistently shown an inverse association between the risk of chronic human diseases such as T2D and the consumption of polyphenolic rich diet (Arts and Hollman 2005).

Our recent study showed that *M. indica* kernel flour is a rich source of pharmacologically important flavonoids (catechin, rutin, quercitrin, quercetin, and kaempferol), and phenolic acids (gallic acid, caffeic, chlorogenic, and ellagic acid) (Irondi et al. 2014). In the same study, we demonstrated that methanol extract of MIKF inhibits some key enzymes (*α*‐amylase, *α*‐glucosidase, and aldose reductase) linked to the pathology and complications of type 2 diabetes (T2D) in vitro. *Mangifera indica* L. (mango) is an important economic tree crop, ranking second as internationally traded tropical fruits, and fifth in total world production among major fruit crops (Food Agriculture Organization FAO 2010). It serves as food and as an important medicinal plant. The fruit which is commonly eaten by man is a rich source of vitamin A, and also contains vitamins B and C; the mesocarp is typically composed of water (84%), sugar (15%), protein (0.5%), fibers and skin (0.5%). The seed kernels contain carbohydrate (80%), fat (10%), and protein (6%) (Purseglove 1991), and are usually discarded as a waste product after the fruit is eaten. However, in India they are consumed by humans during times of food scarcity (Kumar et al. 1991; Purseglove 1991). In the South‐Eastern part of Nigeria, they are used for thickening of traditional soups (Eddy and Udoh 2005). Different parts of the *M. indica* plant are used in folk medicine for a wide variety of remedies (Garrido et al. 2004). The extracts have been reported to have several pharmacological activities such as antioxidant (Ribeiro et al. 2008; Maisuthisakul and Gordon 2009), anti‐inflammatory (Garrido et al. 2004), antidiabetic (Jain et al. 2014), and immune‐modulatory activities (Garcia et al. 2003). This study was therefore designed to evaluate the antidiabetic effects of 10% and 20% *M. indica* kernel flour‐supplemented diet in high‐fat, low‐dose streptozotocin‐induced T2D in rats.

## **Materials and Methods**

### Sample collection and preparation of kernel flour

**Figure 1:**
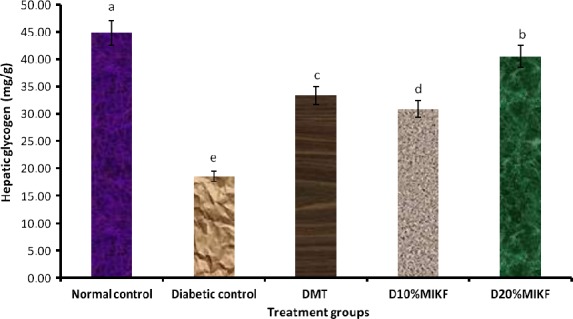
Effect of (MIKF)‐supplemented diets on the hepatic glycogen level of the rats. Bars with different letters differ significantly (*P* < 0.05). DMT, diabetic rats treated with metformin; D10% MIKF, diabetic rats fed 10% *M. indica* kernel flour‐supplemented high fat diet (HFD); D20% MIKF, diabetic rats fed 20% *M. indica* kernel flour‐supplemented HFD.

Samples of fresh ripe *M. indica* seeds (Figure 1) were purchased in a local farm settlement in Ibadan, Oyo State, Nigeria. The seeds were authenticated at the Department of Botany, University of Ibadan, Nigeria. Subsequently, the seeds were sorted and the kernels were manually removed from the endocarp. The kernel flour was prepared by chopping the fresh kernels were with a stainless kitchen knife and sun‐drying for 4 days. Subsequently, the kernels were ground into fine particle size (0.5 mm) using a laboratory grinder to obtain the MIKF. The MIKF was packed in an airtight container and stored at 4°C until analysis.

All the chemicals used for analysis were of analytical grade.

### Determination of proximate composition of MIKF

The proximate composition of the MIKF was determined according to Association of Official Analytical Chemists, AOAC (2005)methods. Moisture content was determined by oven‐drying at 100–105°C for 18–24 h. Total Nitrogen content (*N*) was determined by Kjeldahl method, and the protein content was calculated as *N* × 6.25. Ash content was determined by incinerating 2 g of the MIKF in a preweighed porcelain crucible in a muffle furnace at 600°C for 6 h. Crude fat content of the MIKF was determined using a Soxtec extraction machine. Total carbohydrate was estimated by subtracting the sum of ash, protein, moisture, and fat, from 100 (i.e., 100 – [% ash + % protein + % moisture + % fat]).

### Formulation of experimental diets

The formulation of high‐fat diet (HFD) containing 40% fat (lard) and 18% protein (casein) and 41% carbohydrate (corn starch) as the percentage of total calorie, was based on Reed et al. (2000). Then the HFD was supplemented with 10% and 20% of the MIKF at the expense of corn starch, to give the *M. indica* kernel flour‐supplemented diets (10% MIKF and 20% MIKF diets, respectively). The composition of the experimental diets is shown in Table 1.

**Table 1:** Composition of the experimental diets (g/100 g diet)

Diet	Ingredients (g/100 g diet)					
	Casein	Corn oil	Mineral‐vitamin premix	Lard	MIKF	Corn starch
Basal	19.15	10.00	4.00	–	–	66.85
HFD	19.15	–	4.00	40.00	–	36.85
10% MIKF	18.66	–	4.00	38.63	10.00	28.71
20% MIKF	18.17	–	4.00	37.26	20.00	20.57

MIKF, *Mangifera indica* kernel flour; HFD, high‐fat diet 10% MIKF diet supplemented with 10% *M*. *indica* kernel flour; 20% MIKF; diet supplemented with 20% *M*. *indica* kernel flour.

Casein = 94% protein; lard = 99.9% fat.

### Experimental animals and induction of T2D using the HFD/STZ model

Adult male Wistar rats weighing 180–200 g were used for this study. The rats were procured from the experimental animal breeding unit of Department of Veterinary Medicine, University of Ibadan, Nigeria, and were handled in accordance with the Guide for the Care and Use of laboratory animals of our institution. The rats were housed in experimental cages under a 12 h light‐dark cycle at an ambient temperature of 25 ± 2°C, to acclimatize for 7 days. During the acclimatization period, the rats were fed rodent chow and water ad libitum. To induce T2D, the rats (excluding the normal control rats) were fed the HFD for 2 weeks (Reed et al. 2000), after which they were injected with single dose streptozotocin (STZ) (40 mg/kg b.w., intraperitoneally, in citrate buffer; pH 4.5) (Parveen et al. 2011); the normal control rats were fed basal diet. The development of hyperglycemia in the rats was confirmed by testing for fasting blood glucose (FBG) 72 h after the STZ injection. The rats that maintained FBG higher than 200 mg/dL were considered diabetic, and were selected for the study.

### Animal Groups and experimental design

The rats were divided into five groups of five animals each as follows: group I (normal control), fed‐basal diet throughout the experiment; group II (diabetic control), fed HFD throughout the experiment; group III (DMT), diabetic rats administered metformin (25 mg/kg b.w), whereas on the HFD; group IV (D10% MIKF), diabetic rats fed 10% *M. indica* kernel flour‐supplemented HFD; group V (D20%MIKF), diabetic rats fed 20% *M. indica* kernel flour‐supplemented HFD. The dietary regimen lasted for 21 days, during which FBG was measured at 0, 7th, 14th, 21st day of the study using blood from rats' tail vein. The average food intake and body weight changes of the rats were monitored during the experiment. At the end of the experiment, the rats were fasted overnight, and killed. Then the blood, liver, and pancreas samples of the rats were collected for biochemical assays. The plasma portion of the blood was separated by centrifuging at 800 rpm for 10 min.

### Tissue preparation

The liver and pancreas tissues of the rats were excised immediately after rats were killed, and were perfused with ice‐cold saline. They were later homogenized at 4°C with 10 times w/v 0.1 mol/L phosphate‐buffer, pH 7.4, in a polytron homogenizer. The homogenate was centrifuged at 800 rpm for 5 min at 4°C to separate the nuclear debris, and an aliquot of the supernatant was used for the determination of malonaldehyde (MDA) level.

### Biochemical assays

Fasting blood glucose level was tested using a portable glucometer (Accu Check Active), and the percentage change in FBG level of the rats during the treatment period was calculated. Liver glycogen was determined by the method reported by Ong and Khoo (2000), using anthrone reagent. Glycosylated hemoglobin (HbA 1c) level was determined according to the method of Standefer and Eaton (1983). Plasma lipid profile, namely, triglycerides (TRIG), total cholesterol (TC), and high‐density lipoprotein (HDL) levels were estimated using commercially available standard kits (Randox Laboratories, Ltd., Crumlin, Co. Antrim, UK), according to the manufacturer's instruction. Low‐density lipoproteins (LDL) and very low‐density lipoproteins (VLDL) levels were calculated using the Friedewald formula (Friedewald et al. 1972), as follows:

LDL = TC–(HDL + VLDL)

VLDL = TRIG/5

Hepatic and pancreatic malonaldehyde level as an index of lipid peroxidation was determined according to the method described by Ohkawa et al. (1979). The level of plasma electrolytes (Mg, Zn, and Cu) quantified according to the method reported by Clegg et al. (1981), using atomic absorption spectrometer. Liver function enzymes [plasma aspartate aminotransferase (AST), alanine aminotransferase (ALT), and alkaline phosphatase (ALP)] activities were determined using Randox laboratory kits. Total protein concentration of the plasma and tissues was determined according to the method of Lowry et al. (1951).

### Statistical analysis

Results of replicate experiments were expressed as mean ± standard deviation (SD). Analysis of variance (ANOVA) and least significance difference (LSD) were carried out on the result data at 95% confidence level using SPSS statistical software package, version 17 (SPSS Inc., Chicago).

## Results

The proximate composition of the MIKF is presented in Table 2. The result showed that the MIKF was high in carbohydrate (72.73%), but low in protein (4.59%) and ash (1.69%) contents.

**Table 2:** Proximate composition of the *M. indica* kernel flour (MIKF)

Nutrients	MIKF
Moisture (%)	7.31 ± 0.04
Protein (%)	4.59 ± 0.47
Fat (%)	13.68 ± 0.01
Ash (%)	1.69 ± 0.03
Total carbohydrate (%)	72.73 ± 0.45

Values represent mean ± standard deviation of replicate determinations (*n* = 3).

The average food intake of the rats is presented in Table 3. The results showed that there was no significant (*P* > 0.05) difference in the daily food intake of the various treatment groups. The changes in average body weights (g) of the rats are presented in Tables 4. Although the normal control group had 4.28% body weight gain, the diabetic control group suffered −3.25% loss in body weight; and the diabetic rats fed 10% and 20% MIKF‐supplemented diets had body weight gain of 1.81% and 2.09%, respectively.

**Table 3:** Average food intake of rats fed *M. indica* kernel flour‐supplemented diets

Treatment groups	Average food intake (g/rat/day)
Normal control	5.52 ± 0.46^a^
Diabetic control	6.01 ± 0.47^a^
DMT	5.68 ± 0.42^a^
D10%MIKF	5.88 ± 0.8^a^
D20%MIKF	5.94 ± 0.51^a^

Values represent mean ± standard deviation of replicate determinations (*n* = 5). Values with the same superscript letters along the column are not significantly (*P* > 0.05) different.

DMT, diabetic rats treated with metformin; D10% MIKF, diabetic rats fed 10% *M. indica* kernel flour‐supplemented high fat diet (HFD); D20% MIKF, diabetic rats fed 20% *M. indica* kernel flour‐supplemented HFD.

**Table 4:** Changes in average body weights (g) of type 2 diabetic rats fed soup thickener flour‐supplemented diets

Rat group	Initial weight	Final weight	Weight gain/loss (%)
Normal control	200.88 ± 14.09	209.48 ± 14.12	4.28^a^
Diabetic control	200.62 ± 16.56	194.1 ± 17.02	−3.25^d^
DMT	197.12 ± 14.11	200.04 ± 14.25	1.48^c^
D10%MIKF	204.04 ± 12.29	207.74 ± 12.35	1.81^b^
D20%MIKF	202.88 ± 14.13	207.12 ± 13.86	2.09^b^

Values represent mean ± standard deviation of replicate determinations (*n* = 5). Values with different superscript letters along the column differ significantly (*P* < 0.05).

DMT, diabetic rats treated with metformin; D10%MIKF, diabetic rats fed 10% *M. indica* kernel flour‐supplemented high fat diet (HFD); D20%MIKF, diabetic rats fed 20% *M. indica* kernel flour‐supplemented HFD.

As presented in Table 5, there was significant (*P* < 0.05) increase in the FBG level of the diabetic groups, relative to the normal control group. However, this was significantly (*P* < 0.05) and progressively restored toward normal in the diabetic rats fed MIKF‐supplemented diets, as indicated by the decrease in their FBG levels from the day 0 to the day 21 of the dietary intervention. The diabetic group fed 20% MIKF‐supplemented diet had the highest percentage decrease (−185.28%) in the FBG level.

**Table 5:** Changes in fasting blood glucose (FBG) level (mg/dL) of rats during the dietary intervention

Treatment group	Day 0	Day 7	Day 14	Day 21	% change
Normal control	90.6 ± 5.22^a^	91.8 ± 4.97^a^	91.4 ± 5.46^a^	91.6 ± 4.67^a^	+1.10 (Increase)
Diabetic control	378 ± 7.84^a^	383.6 ± 8.29^a^	392.2 ± 7.40^ab^	394.6 ± 7.73^b^	+4.39 (Increase)
DMT	383 ± 20.29^a^	203.2 ± 16.42^b^	177.2 ± 9.09^c^	146.8 ± 8.17^d^	−61.67 (Decrease)
D10%MIKF	353.2 ± 18.45^a^	234.6 ± 9.42^b^	181.2 ± 6.22^c^	141.2 ± 7.89^d^	−60.02 (Decrease)
D20%MIKF	356.6 ± 15.52^a^	223 ± 9.38^b^	163.8 ± 5.93^c^	125.00 ± 6.82^d^	−64.95 (Decrease)

Values with different superscript(s) within the same row differ significantly (*P* < 0.05).

% change = (FBG at Day 21 − FBG at Day 0) × 100/FBG at Day 0.

DMT, diabetic rats treated with metformin; D10%MIKF, diabetic rats fed 10% *M. indica* kernel flour‐supplemented high fat diet (HFD); D20%MIKF: diabetic rats fed 20% *M. indica* kernel flour‐supplemented HFD.

In Figure 1, the hepatic glycogen level of the diabetic rats decreased significantly (*P* < 0.05) relative to the normal control group. However, the MIKF‐supplemented diets were able to significantly (*P* < 0.05) improve the hepatic glycogen level of the diabetic rats, relative to the diabetic control group. The hepatic glycogen concentration of the diabetic group fed 20% MIKF‐supplemented diet was significantly (*P* < 0.05) higher than those of the groups fed 10% MIKF‐supplemented diet or treated with metformin.

**Figure 2:**
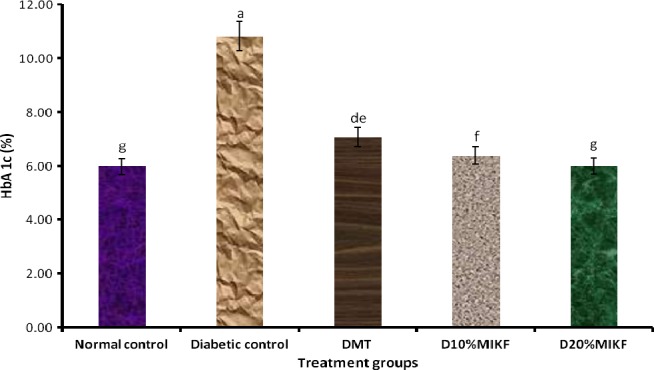
Effect of MIKF‐supplemented diets on the glycosylated hemoglobin (HbA 1c) level of the rats. Bars with different letters differ significantly (*P* < 0.05). DMT, diabetic rats treated with metformin; D10%MIKF, diabetic rats fed 10% *M. indica* kernel flour‐supplemented high fat diet (HFD); D20%MIKF, diabetic rats fed 20% *M. indica* kernel flour‐supplemented HFD.

The level of glycosylated hemoglobin (HbA 1c) of the rats is shown in Figure 2. The diabetic groups had significantly (*P* < 0.05) higher level of HbA 1c than the normal control group. But relative to the diabetic control group, diabetic groups fed MIKF‐supplemented diets had significantly (*P* < 0.05) lower HbA 1c level; indicating that the MIKF‐supplemented diets ameliorated the glycosylation of hemoglobin resulting from the hyperglycemic effect of STZ administration. Interestingly, the HbA 1c level of diabetic rats fed 20% MIKF‐supplemented diet was comparable (*P* > 0.05) with that of the normal control group, but significantly (*P* < 0.05) lower than those of the 10% MIKF‐supplemented diet and metformin‐treated group.

**Figure 3:**
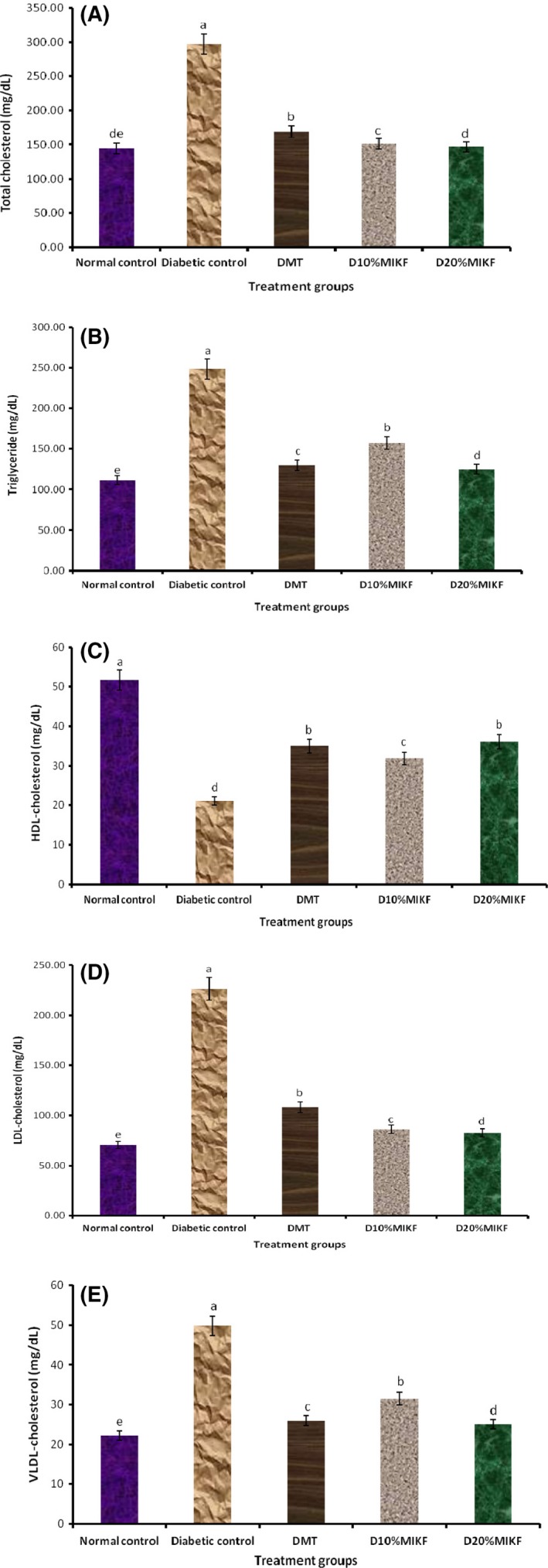
Effect of MIKF‐supplemented diets on the lipid profile of the rats. (A) total cholesterol; (B) triglycerides; (C) high‐density lipoprotein‐cholesterol (HDL); (D) low‐density lipoprotein‐cholesterol (LDL‐C); (E) very low‐density lipoprotein‐cholesterol (VLDL). Bars with different letters differ significantly (*P* < 0.05). DMT, diabetic rats treated with metformin; D10%MIKF, diabetic rats fed 10% *M. indica* kernel flour‐supplemented high fat diet (HFD); D20%MIKF, diabetic rats fed 20% *M. indica* kernel flour‐supplemented HFD.

The effects of the MIKF‐supplemented diets on the lipid profiles [total cholesterol, triglycerides, HDL, Low‐density lipoproteins–cholesterol(LDL‐C), and VLDL of the rats are presented in Figures 3A–E. Relative to the normal control group, the diabetic rats had significant (*P* < 0.05) increase in their plasma total cholesterol, triglycerides, LDL, and VLDL; with a concomitant significant (*P* < 0.05) decrease in their plasma HDL concentration. However, in comparison with the diabetic control group, the diabetic groups fed MIKF‐supplemented diets had significantly (*P* < 0.05) lower levels of plasma total cholesterol, triglycerides, LDL, and VLDL; with an attendant significant (*P* < 0.05) increase in HDL. The 20% MIKF‐supplemented diet was more effective in improving the lipid profile of the diabetic rats than the 10% MIKF‐supplemented diet and metformin.

**Figure 4:**
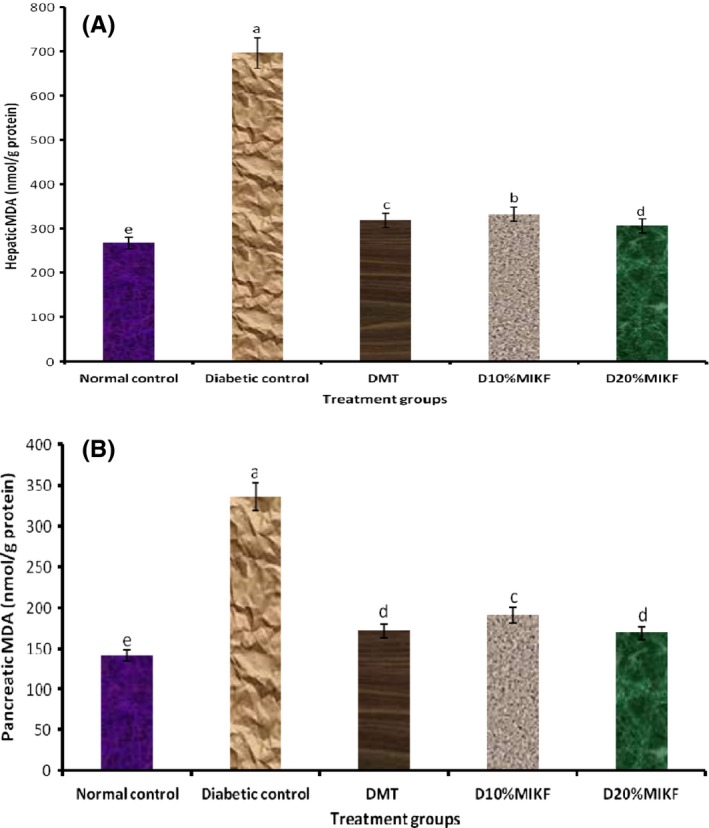
Effect of MIKF‐supplemented diets on the hepatic and pancreatic malonaldehyde level (MDA) of the rats. (A) hepatic MDA; (B) pancreatic MDA. Bars with different letters differ significantly (*P* < 0.05). DMT, diabetic rats treated with metformin; D10%MIKF, diabetic rats fed 10% *M. indica* kernel flour‐supplemented high fat diet (HFD); D20%MIKF, diabetic rats fed 20% *M. indica* kernel flour‐supplemented HFD.

The levels of peroxidation of the liver and the pancreas of the rats, reported as MDA concentration are presented in Figure 4A and B. The diabetic groups had significantly (*P* < 0.05) higher MDA levels than the normal control group. The MDA levels of the diabetic groups fed 20% MIKF‐supplemented diet was comparable (*P* > 0.05) with that of the metformin‐treated group.

The plasma electrolytes [magnesium (Mg), zinc (Zn), and copper (Cu)] concentrations of the rats are presented in Table 6. Mg and Zn decreased significantly (*P* < 0.05) in the diabetic groups compared with the normal control group. However, the plasma Mg and Zn levels of the diabetic groups fed the MIKF‐supplemented diets were significantly (*P* < 0.05) higher than those of the diabetic control group; indicating that the diets were able to improve the plasma Mg and Zn levels of the rats. Conversely, the diabetic groups had significantly (*P* < 0.05) higher plasma Cu level than the normal control group, with the diabetic control group having the highest level.

**Table 6:** Effect of *M. indica* kernel flour‐supplemented diet on the plasma electrolytes concentration of the rats

Treatment group	Mg (mg/dL)	Zn (*μ*g/dL)	Cu (*μ*g/dL)
Normal control	1.99 ± 0.07^a^	149.83 ± 5.53^a^	70.17 ± 3.84^e^
Diabetic control	1.25 ± 0.04^d^	94.17 ± 2.86^d^	128.93 ± 4.56^a^
DMT	1.74 ± 0.05^c^	130.68 ± 4.09^c^	89.70 ± 3.49^c^
D10%MIKF	1.72 ± 0.06^c^	129.73 ± 4.78^c^	98.60 ± 6.61^b^
D20%MIKF	1.89 ± 0.05^b^	142.36 ± 3.89^b^	77.83 ± 5.89^d^

Values represent mean ± standard deviation of replicate determinations (*n* = 5). Values with different superscript letters along the column differ significantly (*P* < 0.05).

DMT, diabetic rats treated with metformin; D10%MIKF, diabetic rats fed 10% *M. indica* kernel flour‐supplemented high fat diet (HFD); D20%MIKF, diabetic rats fed 20% *M. indica* kernel flour‐supplemented HFD.

The alanine aminotransferase, AST and ALP activities (Table 7) were consistently significantly (*P* < 0.05) higher in the diabetic groups than in the normal control group. When compared with the diabetic control group, there was significant (*P* < 0.05) decrease in the activities of these three enzymes in the diabetic groups fed MIKF‐supplemented diets; suggesting that intake of the diets attenuated the level of the hepatocellular damage that might have been caused by STZ administration in the diabetic rats. Relative to the normal control group, there was significant (*P* < 0.05) reduction in the protein level of the diabetic groups. However, intake of the MIKF‐supplemented diets resulted in a significant (*P* < 0.05) increase in the plasma total protein levels of the diabetic groups when compared with the diabetic control group.

**Table 7:** Effect of *M. indica* kernel flour‐supplemented diet on liver function markers of the rats

Treatment group	Alanine aminotransferase (U/L)	Aspartate aminotransferase (U/L)	Alkaline phosphatase (U/L)	Protein (g/dL)
Normal control	23.42 ± 1.01^e^	55.86 ± 1.47^e^	76.91 ± 2.06^d^	7.87 ± 0.13^a^
Diabetic control	51.25 ± 1.65^a^	100.21 ± 2.12^a^	146.88 ± 3.34^a^	3.21 ± 0.11^d^
DMT	30.51 ± 1.16^c^	63.12 ± 1.26^c^	84.14 ± 1.83^c^	5.33 ± 0.21^b^
D10%MIKF	33.60 ± 1.91^b^	66.28 ± 1.91^b^	87.23 ± 2.32^b^	5.15 ± 0.16^bc^
D20%MIKF	27.98 ± 1.46^d^	60.60 ± 1.57^d^	81.68 ± 2.20^c^	5.35 ± 0.22^b^

Values represent mean ± standard deviation of replicate determinations (*n* = 5). Values with different superscript letters along the column differ significantly (*P* < 0.05).

DMT, diabetic rats treated with metformin; D10%MIKF, diabetic rats fed 10% *M. indica* kernel flour‐supplemented high fat diet (HFD); D20%MIKF, diabetic rats fed 20% *M. indica* kernel flour‐supplemented HFD.

## Discussion

In this study, we evaluated the antidiabetic activity of *M. indica* kernel flour (MIKF)‐supplemented diets in T2D rats, having reported in a recent study that MIKF is a rich source of pharmacologically important flavonoids and phenolic acids, and that its methanol extract inhibits some key enzymes (*α*‐amylase, *α*‐glucosidase, and aldose reductase) linked to the pathology and complications of T2D in vitro (Irondi et al. 2014). In view of the adverse side effects and high cost of synthetic antidiabetic drugs, research emphasis has continued to increase on alternative ways of preventing and managing T2D that could be more effective, safer and affordable. Dietary intervention has proven to be one of such ways (Oboh et al. 2010), as the consumption of polyphenol‐rich diets has been reported to protect against development of degenerative diseases including diabetes (Arts and Hollman 2005).

The use of HFD/STZ model to induce T2D in rats has been reported by previous studies (Reed et al. 2000; Parveen et al. 2011). In this model, the HFD leads to hyperinsulinemia, insulin resistance and/or glucose intolerance, and the subsequent STZ administration, a *β*‐cell toxin, results in a severe reduction in functional *β*‐cell mass (Szkudelski 2001; Lenzen 2008). The STZ, a known diabetogen (Marianna et al. 2006), then brings about the destruction of *β*‐cells of the islets of Langerhans, thereby leading to a large‐scale reduction in insulin release. Consequently, the deficiency of insulin causes high glucose level in the blood (hyperglycemia) (Grover et al. 2002) and other metabolic aberrations associated with T2D such as increased cholesterol (Kaur et al. 2011). Usually, hypoinsulinemia is characterized by a reduction in glucose utilization by the tissues, and excessive hepatic glycogenolysis and gluconeogenesis, which all constitute the fundamental mechanism underlying hyperglycemia in DM (Jayasri et al. 2008). Thus, diabetic rats induced using this model show hyperglycemia, hyperlipidemia, and insulin resistance that are similar to the natural history and metabolic characteristics of T2D in human (Srinivasan et al. 2005).

The levels of the proximate parameters of the MIKF (Table 2) are in agreement with the range (moisture: 10.48%; ash: 2.20%; fat: 8.30%, protein: 4.49%; carbohydrate: 72.52%) earlier reported by Eddy and Udoh (2005).

The decrease in the body weight of the diabetic control rats may be due to dehydration and increased muscle wasting (Ene et al. 2007); and ongoing gluconeogenesis, in which fats and proteins are catabolized (Prabhu et al. 2008).

The ameliorative effect of MIKF‐supplemented diets on the hyperglycemic conditions of the diabetic rats observed in this study could be attributed to the flavonoids and phenolic acids present in the flour as earlier reported (Irondi et al. 2014). These phenolic compounds are known to exert their antidiabetic activity through several mechanisms including the inhibition of carbohydrate‐hydrolyzing enzymes (Iwai et al. 2006). The increase in the FBG levels of the diabetic rats was accompanied by a reduction in their hepatic glycogen concentration, relative to the normal control group. Under normal metabolic and physiological conditions, insulin enhances intracellular glycogen deposition by stimulating activities of glycogen synthase and inhibiting glycogen phosphorylase (Shivanna et al. 2013). This regulatory function of insulin is compromised under the hypoinsulinemic condition that is caused by the diabetogenic activity of STZ; this may account for the depleted hepatic glycogen level in the diabetic rats observed in this study. This finding is in conformity with that of Ahmed et al. (2010), who reported a reduction in hepatic glycogen level in STZ‐induced diabetic rats. Interestingly, this aberration was mitigated in the diabetic rats fed MIKF‐supplemented diets, suggesting that there may have been increased insulin release; increased synthesis of glycogen synthase and prevention of its inactivation (Selvan et al. 2008), due to the MIKF. Other researchers have also reported the antidiabetic activity of different parts of *M. indica*. For instance, Muruganandan et al. (2005) reported that the chronic administration of mangiferin at 10 and 20 mg/kg doses, once daily for 28 days, lowered the fasting plasma glucose level of STZ‐induced diabetic rats. Similarly, various extracts of the stem‐bark were reported to possess antihyperglycemic effect in diabetic rats (Ojewole 2005; Bhowmik et al. 2009). On the other hand, the decrease in the FBG level of the diabetic group treated with metformin may be attributed to the antihyperglycemic activities of metformin including increase in insulin sensitivity, enhancement of peripheral glucose uptake, suppression of glucose production by the liver, increase in fatty acid oxidation, and decrease in absorption of glucose from the gastrointestinal tract (Kirpichnikov et al. 2002; Collier et al. 2006).

Glycosylated hemoglobin (HbA1c) is a routine index of T2D, being a biomarker for identifying stress hyperglycemia and diabetic hyperglycemia (Kundu et al. 2013). The glycosylation of hemoglobin occurs progressively and irreversibly during the 90–120 days life span of the erythrocytes (Saudek et al. 2005). It is therefore a more comprehensive measure of total glycemic exposure than FBG due to the representation of blood glucose level in both the postprandial and fasting state (Rohlfing et al. 2000). The increase in HbA1c observed in the diabetic rats relative to the normal control rats may be attributed to the higher rate of glycation of hemoglobin under the hyperglycemic condition occasioned by HFD/STZ administration. This is in line with the reports of cooccurrence of diabetic hyperglycemia with elevated HbA1c by other researchers (Bernadette et al. 2008). However, intake of the MIKF‐supplemented diets led to a reduction in the HbA1c levels of the diabetic rats, when compared with the diabetic control group; this further gave credence to the antihyperglycemic effect of the MIKF.

Dyslipidemia is a common biochemical feature of T2D due to mostly insulin resistance and insulin deficiency (Chahil and Ginsberg 2006), and it is one of the major risk factors for cardiovascular disease (CVD) in DM (Chehade et al. 2013). Abnormalities in lipid metabolism resulting from increased free fatty acid release from insulin‐resistant fat cells leads to alterations in plasma lipid profile in diabetes (Chehade et al. 2013). Under normal metabolic conditions, insulin modulates lipid metabolism by activating lipoprotein lipase, which hydrolyzes triglycerides to release fatty acids and glycerol. Then the fatty acids are oxidized for fuel or reesterified for storage in body tissues. Contrarily, the lipoprotein lipase is inactivated under a condition of insulin deficiency and/or resistance, and this leads to hypertriglyceridemia. The alterations in the plasma lipid profile (elevated triglycerides, total cholesterol, LDL and VLDL; and reduced HDL) of the diabetic rats in this study are in agreement with the alterations in lipid profiles of HFD‐STZ‐induced diabetic rats reported by other researchers (Zhang et al. 2010). Increased LDL level enhances the deposition of cholesterol in the arteries and aorta, and consequently the development of coronary heart disease in diabetic patients, since LDL helps to transport cholesterol from the liver to body tissues (Pedersen 2001). HDL, on the other hand, helps to transport endogenous cholesterol and cholesteryl esters from other body tissues to the liver and steroidogenic tissues where they are metabolized and excreted. Thus, HDL is regarded as a beneficial lipoprotein that prevents cholesterol deposition in the system, thereby preventing atherosclerosis (Xu et al. 2005). The observed decrease in the plasma triglyceride, total cholesterol, LDL, and VLDL; and a concomitant increase in the HDL levels of diabetic rats fed MIKF‐supplemented diets may be due to improved insulin secretion by the islet cells of the pancreas.

Lipid peroxidation is an oxidative deterioration of polyunsaturated lipids, involving reactive oxygen species and transition metal ions, which yields diverse cytotoxic products, most of which are aldehydes, such as MDA (Shalaby and Shanab 2013). MDA and other products resulting from the peroxidation polyunsaturated fatty acids, can disrupt the function of the membrane, increase tissue permeability, and inactivate membrane‐bound receptors and enzymes (Rahman 2003). Excessive lipid peroxidation will in turn cause elevated generation of free radicals, which are harmful to the body cells (Perez‐Gutierrez and Mota Flores 2010). The increased MDA levels of the liver and pancreas of the diabetic rats, when compared with that of the normal control group, indicate that there was oxidative damage (peroxidation) to the lipids of these two key tissues involved in glucose metabolism; and this could be attributed to the hyperlipidemic state of the diabetic rats (Annadurai et al. 2014). However, this was ameliorated in the diabetic rats fed MIKF‐supplemented diets. This observation suggests that the MIKF‐supplemented diets might have the ability to protect the pancreas and liver from oxidative damage.

The reduced plasma concentrations of Mg and Zn, and increased Cu concentration observed in the diabetic groups, relative to the normal control group, is consistent with the findings of Viktorínová et al. (2009), who reported lower levels of plasma Mg and Zn, but higher Cu level in diabetic patients than in normal patients. Lower Zn (Masood et al. 2009) and lower Mg (Diwan et al. 2006) have also been reported in diabetic state in comparison with control subjects. It is well‐known that Zn and Mg enhance insulin function and promote glucose entry into the cells. Masood et al. (2009) stated that the hypersecretion of insulin associated with obese T2D patients could cause hyperzincuria; hence, patients with T2D are more likely to have suboptimal Zn status. Hypomagnesemia in diabetics is attributed to osmotic renal losses from glycosuria, decreased intestinal absorption, and redistribution of Mg from the plasma into blood cells caused by insulin effect (Wälti et al. 2003; Guerrero‐Romero and Rodríguez‐Morán 2006). STZ‐induced diabetes has been reported to increase Cu content associated with the metallothionein of rat liver and kidney tissues (Orhan et al. 2011).

Elevated blood levels of ALT, AST, and ALP indicate liver damage by toxicants or disease condition (Singh et al. 2001), including T2D (Idris et al. 2011). The increase in the activities of ALT, AST, and ALP in the diabetic rats relative to the normal control rats is consistent with the findings of Nwanjo (2007), who also reported an elevation in the activities of liver function enzymes in diabetic rats; and this may be due to necrosis of the liver, and the consequent leakage of these enzymes from the liver into circulation (Mansour et al. 2002). The decrease in the activities of ALT, AST, and ALP in the diabetic rats fed MIKF‐supplemented diets, in comparison with the diabetic control group indicates a possible hepatoprotective effect of the MIKF (Iwalokun et al. 2006).

The reduced plasma protein levels of the diabetic rats, in comparison with that of the normal control rats could be attributed to decreased protein synthesis coupled with increased muscle proteolysis (Gray and Cooper 2011). Insulin regulates protein metabolism by stimulating protein synthesis and retarding protein degradation (Murray et al. 2000). Thus, diabetic hypoinsulinemia results in decreased protein synthesis in all tissues due to decreased production of alkaline phosphatase in absolute or relative deficiency of insulin (Murugan and Pari 2007). The decreased plasma protein was, however, restored toward normal in diabetic rats fed MIKF‐supplemented diets, probably as a result of the potential of the MIKF to improve the secretion, sensitivity and action of insulin in the diabetic rats.

The overall antidiabetic activity of the MIKF may be attributed to its polyphenols, including the flavonoids and phenolic acids, as earlier reported (Irondi et al. 2014). In addition to modulating carbohydrate metabolism by inhibiting *α*‐glucosidase and *α*‐amylase, the polyphenols are known to exhibit antidiabetic effect through other mechanisms such as reduction in intestinal absorption of dietary carbohydrate; improvement of *β*‐cell function and insulin action; stimulation of insulin secretion; and antioxidative and anti‐inflammatory properties (Cabrera et al. 2006; Iwai et al. 2006).

## Conclusion

The *M. indica* kernel flour‐supplemented diets improved the levels of fasting blood glucose, hepatic glycogen, glycosylated hemoglobin, lipid profile, plasma electrolytes, malonaldehyde, and the liver function biomarkers of the diabetic rats. *M. indica* kernel flour could therefore be a promising nutraceutical therapy for the management of T2D and its associated complications.

## Conflict of Interest

None declared.

## Figures and Tables

**Figure A1:**
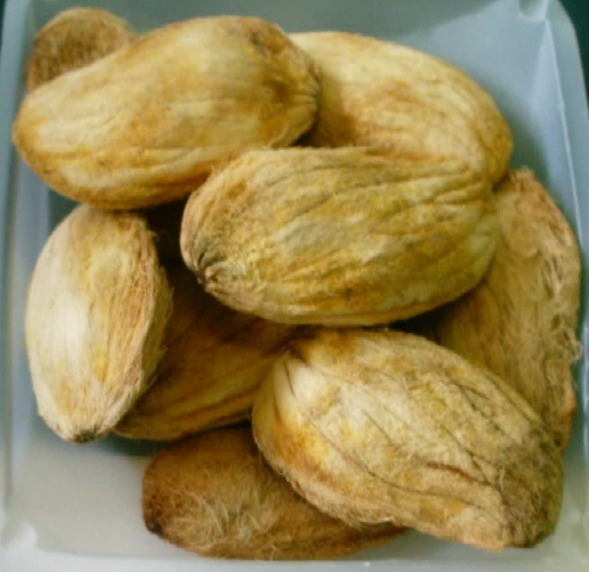
*Mangifera indica* L. (mango) seeds.
